# Iron Transport across Symbiotic Membranes of Nitrogen-Fixing Legumes

**DOI:** 10.3390/ijms22010432

**Published:** 2021-01-04

**Authors:** David A. Day, Penelope M. C. Smith

**Affiliations:** 1 College of Science and Engineering, Flinders University, GPO Box 2100, Adelaide, SA 5001, Australia; 2 School of Life Sciences, La Trobe University, Melbourne, VIC 3083, Australia; P.Smith3@latrobe.edu.au

**Keywords:** legumes, nitrogen fixation, symbiosomes, iron

## Abstract

Iron is an essential nutrient for the legume-rhizobia symbiosis and nitrogen-fixing bacteroids within root nodules of legumes have a very high demand for the metal. Within the infected cells of nodules, the bacteroids are surrounded by a plant membrane to form an organelle-like structure called the symbiosome. In this review, we focus on how iron is transported across the symbiosome membrane and accessed by the bacteroids.

## 1. Introduction

Iron is an essential nutrient for cell function and plants have developed specialised mechanisms for mobilising, absorbing and storing the metal. Cellular iron homeostasis is important because iron is toxic when present in excess. Symbiotically grown legumes have a particularly high requirement for iron and low iron severely retards their growth and their ability to fix atmospheric nitrogen [1,2].

**Figure 1:**
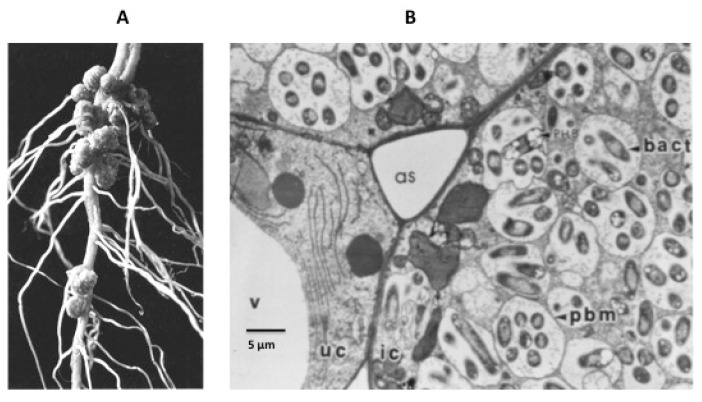
(**A**) nodules on the root of a soybean plant. (**B**) detail of infected cells. V = vacuole; as = air space; uc = uninfected cell; ic = infected cell; pbm = peribacteroid membrane, now known as the symbiosome membrane; bact = bacteroid.

Legumes form a symbiosis with nitrogen-fixing soil bacteria (rhizobia) that enables the plants to utilize atmospheric nitrogen for growth. Infection of the legume root by rhizobia results in the formation of specialized organs called nodules that provide the microaerobic conditions required for operation of the bacterium’s nitrogenase enzyme. The establishment of the nodule begins with signaling between the plant root and the rhizobia, and results in the bacteria colonizing root hairs. An infection thread is formed from invagination of the root cell wall and the bacteria travel through the infection thread into the root cortex where nodule formation is initiated. Eventually, in response to unknown signals, the wall of the infection thread and that of root cortex cells are degraded and the rhizobia released into the cells. Within the nodule infected cells, the rhizobia are enclosed in a plant-derived membrane to form an organelle-like compartment called the symbiosome (Figure 1). Within this symbiosome the rhizobia differentiate into their symbiotic form, the bacteroid, which begin to fix nitrogen to ammonia via the enzyme nitrogenase. The symbiosome membrane (SM) effectively controls the exchange of metabolites between the symbiotic partners. The SM, although originating from the plasma membrane of infected nodule cells, evolves over the course of nodule organogenesis to become a new and specialized membrane containing symbiosis-specific proteins synthesized from plant nuclear genes (see [3,4,5] for recent reviews).

Mature nodules are composed of an infection zone, which contains both infected and uninfected cells, surrounded by layers of cortical cells (3,5). Nutrients are transported to the nodule via the vasculature, which terminates in the cortex. Nodules are generally considered as two distinct types, determinate and indeterminate. Determinate nodules, such as those on soybean and *Lotus japonicus*, are spherical and clearly divided into the central infected zone and the outer cortex. Cells in the infected zone contain bacteroids all at approximately the same developmental stage. Indeterminate nodules, on the other hand, have an elongated or branched shape and have a persistent meristem that remains throughout the life of the nodule. Indeterminate nodules are segmented into distinct developmental zones: a meristematic zone at the tip, an invasion zone where the invading rhizobia are released, a transition zone where the rhizobia differentiate into bacteroids, a nitrogen fixation zone, and a zone of senescence closest to the root. 

Nitrogen fixing legume nodules have a very high requirement for iron and nodule iron content is higher than any of the other plant organs [4,6,7,8]. Iron is an essential cofactor for nitrogenase, the nitrogen-fixing enzyme, as well as respiratory chain components and other essential enzymes in bacteroids [9]. It is also required for the infected cells’ mitochondrial respiratory chain components and for the synthesis of leghemoglobin in the infected cells, which is essential for the transport of oxygen to the bacteroids [8,9,10]. Given that leghemoglobin is present in mM concentrations in the infected cell cytosol [10,11] and that infected cells in a mature soybean nodule contain up to 12,000 mitochondria per cell [12] and as many as 50,000 bacteroids [13,14], then the requirement for iron in nodule cells is enormous. 

Nitrogen fixation has been studied extensively in a number of different legumes, including the model plants *Medicago truncatula* and *Lotus japonicus*, and important crops such as soybean and pea. Iron is most likely imported into the nodule as ferric citrate via the xylem [15,16] and subsequently must cross a number of cell layers to reach the infected cells [17]. Both symplastic and apoplastic routes are utilized for this transport [15]. A member of the NRAMP family in *M. truncatula*, MtNRAMP1, catalyses ferrous iron uptake into infected cells [18], possibly acting in concert with Multidrug and Toxic Compound Extrusion protein 67 (MtMATE67), which catalyzes citrate efflux and appears to enhance iron uptake by infected cells [19]. In this review, we focus on the subsequent events during which iron within nodule infected cells is transported into the symbiosomes for delivery to the bacteroids.

## 2. Studies with Isolated Symbiosomes

Most studies have utilised soybean nodules because of the relative ease of symbiosome purification from this species. Initial experiments showed that isolated symbiosomes accumulate ferric iron when presented with ferric citrate [20,21], and that this involves the concomitant uptake of citrate [21]. However, the iron was not taken up by the bacteroids within and remained in the symbiosome space [20]. Another study showed that soybean symbiosomes contain a large amount of non-heme iron apparently bound to siderophores released from the bacteroids into the symbiosome space [22]. A subsequent study showed that isolated symbiosomes could also take up ferrous iron, which was accumulated by the bacteroids [23]. Isolated soybean symbiosomes possess ferric chelate reductase (FRO) activity on the SM [20] and although the orientation of the enzyme was not ascertained, by analogy with the role of FRO on the plasma membrane it is likely that FRO on the SM oxidises NADH in the plant cytosol and reduces ferric iron in the symbiosome space to provide ferrous iron that could then be taken up by the enclosed bacteroids [24].

The picture emerging from these early studies is that symbiosomes can access both ferric and ferrous iron from the plant infected cell cytosol, that bacteroids prefer ferrous iron, and that the symbiosome space is a storage compartment for iron. In this respect the symbiosome plays a role similar to that of the vacuole, which is an iron storage compartment in other plant cells, and in this context we note that symbiosomes effectively replace the vacuole of infected cells [25,26]. The energetics of the symbiosome also resemble that of the vacuole, with an acidic interior and a positive inside membrane potential, but this is set up and maintained by a P-type ATPase on the SM [27,28,29].

## 3. Iron Transporters Targeted to the Symbiosome Membrane

### 3.1. ZIP and NRAMP Homologues in Nodules

Initial attempts to identify the plant genes encoding symbiosome iron transporters used nodule cDNA libraries to search for homologues of known iron transporters in other plant species. A soybean homologue of the ZIP (ZRT, IRT-like) protein family, GmZIP1, was identified and shown to reside on the SM [30]. ZIP proteins transport a number metal ions including iron [31], but GmZIP1 is relatively specific for zinc and does not transport iron. A member of the Natural Resistance-Associated Macrophage Protein (NRAMP) metal ion transporter family, Divalent Metal Transporter (GmDMT1), was also localised to the SM in soybean and shown to complement an iron-transport deficient yeast mutant [32]. However, the fact that GmDMT1 transports iron into the cytoplasm when expressed in yeast together with the known biochemistry of other NRAMP family transporters [33], suggest it is unlikely to transport iron into the symbiosome (which is equivalent to export from the cytoplasm) [7,16]. GmDMT1 is phylogenetically most similar to *Arabidopsis thaliana* NRAMP3 and NRAMP4 that are localized on the tonoplast and remobilize stored iron during germination [34,35]). Given that symbiosomes effectively replace the vacuole in infected cells and contain stored iron (see above), GmDMT1 is more likely to transport iron out of the symbiosome to the plant cytoplasm [7,16].

### 3.2. Vacuolar Iron Transport Homologues in Nodules

Vacuolar iron transport (VIT) proteins and the closely related VIT-like (VTL) proteins are well characterized in yeast, plants and *Plasmodium*, mediating iron transport into the vacuole for detoxifying excess iron, or for building up iron stores in seeds [36]. The first VIT gene, CCC1, was identified in *Saccharomyces cerevisiae*, where it is required for the transfer of ferrous Fe from the cytosol to the vacuole [37]. In *Arabidopsis* and wheat, VIT proteins also export iron out of the cytoplasm into vacuoles [36,38,39].

Two nodule homologues of VIT proteins, Nodulin-21 in soybean and SEN1 in *Lotus japonicus*, were identified some time ago, but have only recently been placed in context. Nodulin 21 is expressed specifically in nodules of symbiotic plants [40] and in *L. japonicus*, the *sen1* (stationary endosymbiont nodule) deletion mutant is unable to fix nitrogen [41,42]. Transcriptomic data for soybean [43,44] identified another member of the VIT family with high expression in nodules, with very high (88%) identity to Nodulin 21 [16]. These two proteins have subsequently been renamed GmVTL1a and 1b [45,46]. Homologues of GmVTL1 have been characterized in *M. truncatula*: MtVTL4 and 8 are nodule expressed VTLs with MtVTL8 localised on the symbiosome membrane [47]. Their genes are arranged as part of a tandem array of five VTL genes [47].

GmVTL1a, 1b and MtVTL8 all transport ferrous iron when expressed in yeast and are localized to symbiosomes in nodule infected cells [46,47]. In soybean a *vtl1* mutant (knockdown of VTL1a and 1b) and in *M. truncatula* a mutant with a deletion of the VTL tandem array, were compromised for nitrogen fixation and nodule development, and the iron content of nodules and bacteroids was reduced [45,47]. This phenotype was almost completely restored by expression of GmVTL1a or MtVTL8 in the respective mutants, demonstrating their importance in nodules compared to the other VTL genes. Although GmVTL1b could transport iron (although less efficiently than GmVTL1a) in yeast [46], it was not able to restore nitrogen fixation in the *vtl1* mutant [47], suggesting it plays a different role from that of VTL1a. GmVTL1a is also able to rescue the *Ljsen1* mutant, restoring growth and symbiotic nitrogen fixation, suggesting that SEN1 is also localized on the symbiosome membrane [46]. Taken together, the results from these three separate studies indicate that SM-localised VTL proteins are the major routes by which iron is transported into symbiosomes of legume nodules.

### 3.3. Ferroportins

Ferroportins are iron-binding proteins that catalyse ferrous iron efflux from the cytosol of root cells, either out of the cell (eg into the xylem) or into internal organelles, including the vacuole [48,49]. Recently, Escudero et al [50] identified the ferroportin protein MtFPN2 in *M. truncatula*; it is expressed in nodule vascular tissue as well as in the infected zone where it is localized to the symbiosomes. Expression of MtFPN2 in yeast showed that it is an iron efflux protein. Loss of function mutants have disturbed nodule iron distribution and decreased nitrogen fixation ability, which can be restored by expression of the functional protein only in the infected cells. Escudero et al [50] suggest that MtFPN2 is an SM ferrous iron transporter that may work in conjunction with the citrate transporter MtMATE67, which is also located on the SM and is essential for the symbiosis [19]. As discussed earlier, isolated symbiosomes take up both iron and citrate from a solution containing ferric citrate [21], and coordinated operation of both FPN and MATE proteins could account for this, but so too could operation of VTL together with MATE. 

Detailed proteomic analyses of soybean SM [51] have found peptides for MATE, suggesting that it is a common feature of legume symbiosomes, but FPN peptides have not been detected. It is worth noting also that loss of function of VTL in the *Ljsen1* mutant completely blocks nitrogen fixation, so ferroportin may not be a general feature of all legumes. There are many differences in both structure and metabolism between determinate and indeterminate nodules and so differences in iron transport mechanisms are not surprising. Studies with a wider range of species are needed.

Why *M. truncatula* needs two mechanisms (MtVTL and MtFPN) for the transport of ferrous iron across the symbiosome membrane is not immediately apparent, especially since interference with the expression of either protein decreases nitrogen fixation [47,50]. It is possible that the two systems are differentially regulated or that they are expressed at different time points in nodule development and maturation. It would be interesting to compare the effect of a double mutation on symbiotic nitrogen fixation.

### 3.4. Ferrous Versus Ferric Iron Transport across the Symbiosome Membrane

All of the iron transporters discussed above catalyze ferrous iron movement. Yet two independent studies have shown that isolated symbiosomes can accumulate ferric iron [20,21]. The most widely studied ferric iron transporters in plants are the YSL (Yellow Stripe Like) proteins [52,53], part of the larger Oligopeptide Transport (OPT) family. Nodule-enhanced YSL proteins have been identified in both soybean [51,54] and *M. truncatula* [55]. GmYSL7 is localised to the SM, while MtYSL7 is located in the nodule cortex, and interference with their expression compromises nodule development and nitrogen fixation and, in the case of MtYSL7, iron distribution in the nodule. However, neither protein transports iron but rather transport small peptides when expressed in yeast. Whether their effect on nodule iron homeostasis is direct or a result of delayed nodule development remains to be determined, but they are not candidates for the observed ferric iron uptake across the SM. It is possible that the VTL proteins on the SM transport both ferrous and ferric iron and, in this context, it should be noted that the *Ljsen1* mutants with missing or non-functional VTL protein in *L japonicus*, completely lack nitrogen fixation [41,42]. Assuming that *L. japonicus* has similar systems for iron uptake into symbiosomes as soybean, this suggests that VTL is the sole transporter for iron into symbiosomes in *L. japonicus* and that it transports both ferrous and ferric iron.

### 3.5. Iron Import into Bacteroids

Once iron has entered the symbiosome, it must be imported into the enclosed bacteroids. Studies with isolated symbiosomes suggest the preference is for ferrous iron. A recent study has shown that in soybean, feoA and feoB proteins are responsible for ferrous iron uptake into bacteroids [56], and strains with deletions of either gene produce small nodules with few bacteroids. However, a feoA (E40K) mutant with reduced iron uptake activity formed functional nodules, which allowed characterization of iron transport in isolated bacteroids and in whole nodules, showing that the defect did not affect iron provision to nodules even though it reduced uptake into bacteroids. The study also supported the notion that it is ferrous iron that is imported into bacteroids with ferric iron reduced, most likely via a plant ferric reductase, before uptake. It is likely the *FeoAB* genes were not identified in mutant screens because they are also required in free-living bacteria, in both iron-replete and iron deficient conditions, and so would not grow in the culture conditions used to propagate mutants before inoculation of soybean. FeoB encodes a transmembrane protein with a G-protein domain that mediates transport of ferrous iron [57] while FeoA encodes an auxillary protein that is required although the exact function is not clear. While *FeoAB* operons are present in a range of prokaryotes they are not present in all rhizobia strains, including *Sinorhizobium meliloti* and *Rhizobium leguminosarum*. This suggests that there is a yet undiscovered mechanism for import of iron into the bacteroids of these rhizobia and other α-proteobacteria [58].

## 4. Summary

The picture of iron transport within the infected cells of nitrogen-fixing legume nodules has become clearer with the publication of a number of recent studies with soybean and *M. truncatula*, but some questions remain, particularly with respect to ferric iron transport. We also need further investigation of the apparent differences between indeterminate and determinate nodules, such as the presence of ferroportin and the localization of YSL7 proteins.

**Figure 2:**
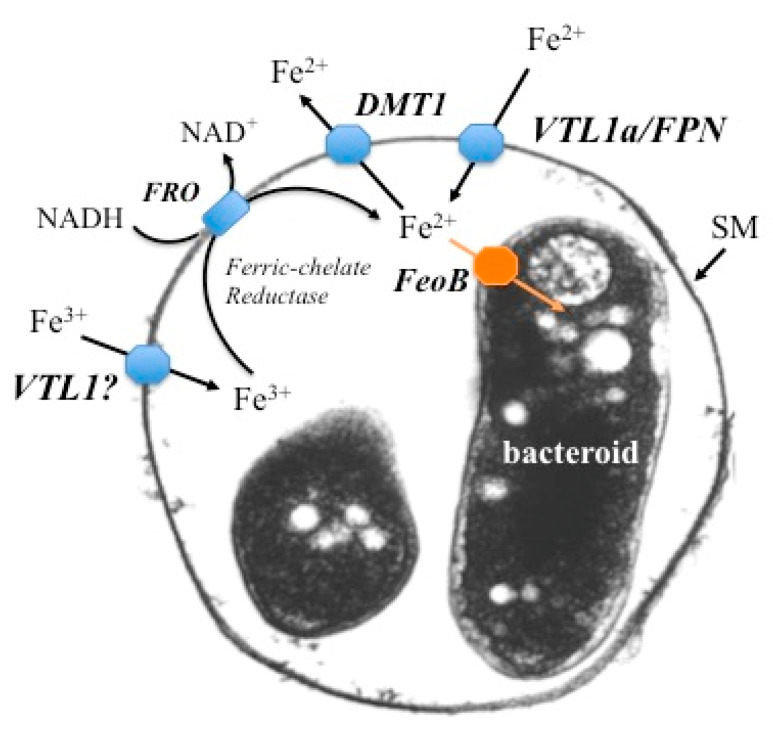
Schematic representation of proposed iron transport in a symbiosome. A symbiosome membrane (SM) surrounding two bacteroids is shown. Ferrous iron is transported across the symbiosome membrane via two avenues: VTL1a (Vacuolar iron Transporter Like protein) in soybean and *M. truncatula* and FPN (ferroportin) in *M. truncatula*. Ferric iron is also transported into the symbiosome, but the protein responsible has not been identified. The symbiosome space is a storage compartment for iron, in much the same manner as a vacuole. Ferric iron in the symbiosome space can be reduced by a ferric chelate reductase (FRO) on the SM and transported either into the bacteroid via the freoB transporter or back into the infected cell’s cytosol via DMT1 (Divalent Metal Transporter). See text for more details.

Figure 2 summarizes our current understanding. A number of candidate proteins on the SM for ferrous iron transport into the symbiosome have been identified and shown to be essential for the symbiosis. The symbiosome appears to be an iron storage compartment for infected cells, containing large quantities of non-heme iron. The iron stored within the symbiosome can be transported into the bacteroid, most likely as ferrous iron and catalyzed by the FeoA and FeoB proteins, at least in soybeans [56], or exported back into the infected cell via DMT1. We suggest that in this regard, the symbiosomes function as vacuoles in buffering iron concentrations. This allows the infected cells to accumulate large quantities of iron to accommodate their large demand, while avoiding iron toxicity in their cell cytoplasm and in the bacteroids. However, it should be noted that iron storage in symbiosomes has only been reported for soybean nodules, which are the determinate type. The picture in indeterminate nodules is less clear and we need further studies in a wider range of legumes.
